# Influence of Work on Elevated Blood Pressure in Hispanic Adolescents in South Texas

**DOI:** 10.3390/ijerph16071096

**Published:** 2019-03-27

**Authors:** Eva M. Shipp, Sharon P. Cooper, Luohua Jiang, Amber B. Trueblood, Jennifer Ross

**Affiliations:** 1 Texas A&M Transportation Institute, Center for Transportation Safety, 2929 Research Parkway, College Station, TX 77843, USA; a-trueblood@tti.tamu.edu; 2 Texas A&M University School of Public Health, Department of Epidemiology & Biostatistics MS 1266, College Station, TX 77843, USA; 3 The University of Texas Health Science Center at Houston–San Antonio Regional Campus; sbpcooper@yahoo.com; 4 Department of Epidemiology, University of California, Irvine, CA 92697, USA; lhjiang@uci.edu; 5 University of Oklahoma, College of Arts & Sciences, 633 Elm Avenue, Norman, OK 73019, USA; jenross@ou.edu

**Keywords:** adolescent, hypertension, blood pressure, Hispanic, work, farmworker, occupational health

## Abstract

Literature supports an association between work and cardiovascular disease in adults. The objective of this study was to examine the relationship between current work status and elevated blood pressure in Hispanic adolescents. Participants were students in Hidalgo County, located along the Texas-Mexico border. Participants enrolled in the cohort study in ninth grade with assessments completed once a year for up to three years. Participants completed a self-report survey, while staff measured height, weight, waist circumference, blood pressure, and were screened for acanthosis nigricans. A generalized linear regression model with a logit link function was constructed to assess current work status and elevated blood pressure. Of the 508 participants, 29% had elevated blood pressure, which was associated with being male and other chronic disease indicators (e.g., acanthosis nigricans, overweight/obesity). The mean probability for elevated blood pressure was higher among currently working adolescents compared to those who were not. Findings were statistically significant (*p* < 0.05) at baseline. The findings illustrate that a large proportion of adolescents along the Texas-Mexico border may have elevated blood pressure and that working may be associated with it. Subsequent research is needed to confirm these findings, as well as to identify the mechanism for how work may increase hypertension in adolescents.

## 1. Introduction

High blood pressure is among the most important risk factors for cardiovascular disease (CVD), the leading cause of death in adults, and the fifth leading cause of death among those aged 15–24 years in the United States [1,2,3]. Based on cross-sectional data from the National Health and Nutrition Examination (NHANES) in 2013–2014, an estimated 31.6% of adults aged 18 years and older were hypertensive, which amounted to approximately 75.1 million adults. Only about half of these adults (53.9%) had their blood pressure under control [4], leaving them especially vulnerable to CVD and related health problems.

Hypertension is a critical public health issue that may originate to some extent in childhood or adolescence [1,5,6,7,8]. As an example, high blood pressure during adolescence is associated with persistent cardiovascular alterations that can continue into adulthood [1,7,8]. In addition, national survey data illustrate that approximately one in ten adolescents screen positive for elevated blood pressure across the United States. Based on NHANES data from 2011–2012, elevated blood pressure, defined as high or borderline, was reported for 11% of those aged 8–17 years [9]. The prevalence was higher among males (15.4%) versus females (6.8%), Hispanics (11.5%) and non-Hispanic blacks (15.3%) versus non-Hispanic whites (9.4%), and those 13 to 17 years of age (15.0%) versus those 8–12 years of age (6.5%) [9]. In 2017, the American Academy of Pediatrics put forth revised clinical practice guidelines as an update to the 2004 “Fourth Report on the Diagnosis, Evaluation, and Treatment of High Blood Pressure in Children and Adolescent” [10]. Although the new guidelines are not a substantial departure from the prior report, applying the criteria to NHANES and other data suggests that more children would be classified as having elevated blood pressure and that shorter children aged 13 years and younger, and children over 13 years of age of any height may have a greater likelihood of being diagnosed as hypertensive [11,12]. Sharma and colleagues analyzed NHANES data from 1999–2014 and found that the prevalence of elevated blood pressure among children aged 5–18 years was 11.8% under the previous guidelines versus 14.2% under the revised guidelines [12].

Along with age, race and ethnicity, it is well known that socioeconomic status, lifestyle factors, nutrition, exercise, and body composition (e.g., excess body fat) play important roles in the occurrence and prevention of hypertension in adults [13,14,15]. A growing number of studies provide evidence that adolescents with elevated blood pressure also may be more likely to consume higher levels of sodium, lower levels of potassium, and more dietary fat; be less physically active; have poorer sleep quality; and be from lower socioeconomic levels [9,16,17,18,19,20,21,22,23]. However, the relationships between potential risk factors and the magnitude of their association with elevated blood pressure have not been established conclusively in younger populations.

Work-related factors are also consistently associated with hypertension in adults. Specific issues associated with increased blood pressure include job insecurity, long work hours, low wages, and jobs with poor work organization, as defined by jobs with high demands with low control or jobs that require considerable effort with low reward [24,25,26,27,28]. The exact mechanisms governing how these issues increase blood pressure is not entirely known. In adult workers, work stress may result in repeated activation of the autonomic nervous system, which can contribute to high blood pressure and heart disease based on studies with adult workers [29]. In addition, the time spent working may simply decrease the amount of time available for physical activity or other healthy behaviors (e.g., healthy diet). This may be especially true for adolescents who already contend with notable time demands, including attending school, participating in after school activities including team sports with considerable practice requirements, and helping with family chores and other obligations. Finally, work stress also may increase unhealthy coping behaviors such as excessive food consumption, consuming foods higher in sugar and saturated fat, or consuming alcohol. For example, a study found that fast food restaurant use among adolescents was associated with student employment, television usage, perceived barriers to healthy eating, and availability of unhealthy foods [30]. In addition, studies on working adolescents illustrate that long work hours contribute to the early onset of alcohol use [31,32,33,34].

The National Institute for Occupational Safety and Health (NIOSH) has recognized for over a decade that a variety of work-related exposures and issues influence overall health and well-being. From this perspective, whether and how work-related factors including stress, workload, and work hours influence health conditions (such as chronic disease) is as important as ensuring a safe workplace in a more traditional sense (e.g., minimizing physical hazards to address acute injuries). In 2011, NIOSH put forth the Total Worker Health Program to continue elevating this issue, spur further research, and promote workplace wellness programs [35]. In line with this holistic approach to worker health, the objective of the present study was to begin examining the relationship between current work status and elevated blood pressure in adolescents.

## 2. Materials and Methods

### 2.1. Study Design

Data for the present study, a secondary analysis, were collected during a prospective cohort study that was designed to estimate the occurrence of chronic disease indicators among students enrolled in a Migrant Education Program (MEP) and a comparison group of non-MEP students [36]. Students qualified for MEP if he or she migrated or had at least one parent who migrated within the prior three years to work in agriculture or fishing as a principal means of employment. “Migrated” was defined as moving temporarily to a different school district or administrative area within the United States [36]. All students enrolled in ninth grade and in MEP in two public high schools in Hidalgo County, Texas (located along the Texas-Mexico border) were recruited to participate and an equal number of students in the ninth grade who were not enrolled in MEP were randomly sampled as a comparison group. Enrollment occurred in 2007 and 2008 with participants followed for up to an additional three years (2008–2010). At the baseline and follow-up assessments, participants completed a survey on demographics, health behaviors, and work characteristics. A minimum clinical examination was also completed, which included measured height, weight, and waist circumference, a noninvasive visual screening for acanthosis nigricans (AN) on the neck, and blood pressure. Prior to partaking in the study, both parental and participant written consent was obtained. Data collection took place each year from January to March to accommodate the migration schedule of MEP students. Data collection took place in a private area in each school during approximately one class period. All interviewers were bilingual, certified in ethical standards for research with human subjects, and retrained in study methods each year to promote adherence to data collection protocols throughout the duration of the study. Additional methodological details are provided by Cooper and colleagues (2016) [36].

This study was approved by the Institutional Review Boards at The University of Texas Health Science Center at Houston (HSC-SPH-07-0284) and Texas A&M University (2010–0878). Written informed consent from parents and written child assent was obtained prior to any data collection.

### 2.2. Sample

A total of 628 students (n = 297 MEP; n = 331 non-MEP) were asked to complete a baseline assessment during the first and second year of recruitment. Of those, 508 enrolled (257 MEP and 251 non-MEP) and participated in the baseline assessment for a response proportion of 80.9%. For the follow-up assessments, participants with a baseline assessment who were still enrolled in school were eligible to participate. Of those, greater than 90% participated in the follow-up assessments each year. The available sample size for each study year were as follows: baseline (n = 257 MEP, n = 251 non-MEP), follow-up year 1 (n = 209 MEP, n = 220 non-MEP), follow-up year 2 (n = 165 MEP, n = 181 non-MEP), and follow-up year 3, which only included participants who enrolled during the first recruitment year (n = 65 MEP, n = 65 non-MEP). This study focused on the baseline and follow-up years 1 and 2 only.

### 2.3. Variable Definitions

Variable definitions and data collection protocols included the following. Staff measured blood pressure using an automated device, the Omron HEM-907XL (Omron Healthcare, Lake Forest, IL, USA). This ensured measurement consistency across participants and across survey years. Staff applied the cuff to students’ right arm with measurements taken on a single occasion. After five minutes of quiet rest, the automated device took three successive measurements, while the participant sat in a chair with back support, feet flat on the floor, and arm supported with the antecubital fossa at heart level [36]. Staff recorded the first measurement, but it was not included in analysis, since these measurements can be falsely elevated [36]. Consequently, all analyses included only the average of the second and third measurements [36]. The definition of elevated blood pressure was at or above the 90th percentile for age, height, and sex or ≥120/80 mm Hg [36]. Staff recorded height to the nearest 0.1 cm using a Shorr Board stadiometer (Shorr Productions, Olney, MD). Staff measured weight using a portable Tanita BWB-800S digital scale that was certified to be accurate to 400 pounds each year by a professional scale service and maintenance company (Tanita Corporation, Arlington, IL, USA). Overweight or obese categories were based on a body mass index (BMI) at or above the 85th percentile for age and sex, which follows the BMI-for-age- weight status categories provided by the Centers for Disease Control and Prevention (CDC) [37]. BMI was calculated as weight in kilograms divided by the height in meters squared. Waist circumference was measured to the nearest 0.1 cm using a plastic tape measure that was stretch resistant. A waist circumference at or above the 75th percentile for age, sex, and ethnicity was defined as abdominal obesity [36]. AN is a dark discoloration and/or thickening of the skin the back of the neck that is used as an indicator of high insulin levels or resistance. Staff used a similar approach to assess AN as those implemented in a Texas state-wide school- screening program [36,38,39]. The basis of work status was a self-reported annual work history of job type, dates of employment, and number of hours worked with survey items modelled after prior studies with working youth [40,41]. The recall period began from January 1st of the year prior to data collection with data collection occurring between January through March each year. The definition of current work status was working for pay or not for pay. The current work status definition was a student who reported a having a job during the same week when they participated in the survey. The basis of items pertaining to health behaviors was the Youth Risk Behavior Surveillance System YRBSS [42].

### 2.4. Data Management and Analysis

Data were managed using a Microsoft SQL relational database (Microsoft, Redmond, WA, USA). All data were double entered into the database to minimize data entry errors, as well as computer edited for out-of-range and contradictory values. SAS 9.4 was used for all data analysis (SAS Institute Inc., Cary, NC, USA). Descriptive statistics included means and proportions at baseline and years of follow-up. Appropriate statistical tests including chi-square tests, t-tests, and Fisher’s exact tests were used to compare the distribution of variables across the two schools and by elevated blood pressure status. Generalized linear mixed models with a logit link function and binomial distribution were used to estimate the probability of high or high normal blood pressure at each time period by current work status after adjusting for potential confounders, including age, gender, AN, BMI, number of days physically active in the past 7 days, and school enrolled. Random intercepts at the individual level were also included in these models to account for potential correlation of repeated measures from the same student. Statistical significance was set a priori at a level of alpha <0.05.

## 3. Results

### 3.1. Participants

Table 1 presents baseline characteristics of participants stratified by school. Overall at baseline, half of participants were male with a mean age of 15.0 years. The place of birth for the majority of participants was the United States (92%). As previously reported in Cooper et al. (2016), a majority of participants self-identified as Hispanic, Latino, or Mexican-American (97%) and used English to complete the survey (79%) (data not shown) [36]. A larger proportion (62%) were enrolled in School 2 than School 1. At baseline, the distribution of demographic and other variables was similar in Schools 1 and 2 with two exceptions. School 2 had a larger percentage of participants who worked in the previous year compared to School 1, 54% and 67%, respectively (*p* = 0.01). However, the prevalence of being currently employed was not statistically different at baseline. School 2 also had a larger percentage of participants who had elevated blood pressure compared to School 1, 33% and 23%, respectively (*p* = 0.01).

### 3.2. Elevated Blood Pressure

Overall, 29% of the participants had elevated blood pressure. Table 2 presents the distribution of baseline characteristics by school and elevated blood pressure status. For School 1 and School 2, elevated blood pressure was more common among males and those with chronic disease indicators including AN, overweight or obesity, and abdominal obesity. Differences were statically significant (*p* < 0.05). Additional statistically significant (*p* < 0.05) differences for School 1 included increasing days physically active in the past seven days and for School 2, increasing participation in the number of sports team during the past 12 months, participation in work in the previous year as well as current employment status.

### 3.3. Current Work Status

At each survey period, the prevalence of current work status at baseline and first and second years of follow-up was 4.3%, 7.0%, and 6.9%, respectively. At baseline, 26 jobs were held by 22 participants. Job types included farm work (n = 3), adult or child care (n = 7), restaurant waiter or cashier (n = 4), lawn care (n = 3), construction (n = 2), office work (n = 2), fast food cashier or worker (n = 1), general cashier or sales (n = 1), grocery stocker or cashier (n = 1), skilled labor (n = 1), and other (n = 1). During the first and second years of follow-up, similar jobs were held. The most common jobs accounted for 70% of job types and included fast food cashier or worker restaurant waiter or cashier, general cashier or sales, grocery store stocker or cashier, and yard work. On average at their current job, participants engaging in farm work reported working 6.5 hours per day on work days (range: 2–12) and 5.8 days per week (range: 1–7), while participants engaging in non-farm work reported working 5.3 h per day on work days (range: 1–13) and 3.6 days per week (range: 1–7). Work intensity was similar across all three assessment years.

### 3.4. Work Status and Elevated Blood Pressure

Table 3 presents the results of the statistical model constructed to estimate the association between work status and elevated blood pressure at baseline and the first and second years of follow-up. Based on variables associated with elevated blood pressure at the bivariate level from Table 2, the following were included in the model as potential confounders: school, gender, age, AN, BMI, and number of days physically active in the past seven days. The adjusted mean probability of having elevated blood pressure at baseline among currently working adolescents was approximately twice the probability observed for adolescents who were not currently working. The difference in adjusted mean probability between the two groups was 27% and statistically significant (*p* = 0.01). Although the differences in the adjusted mean probabilities for follow-up year 1 and follow-up year 2 were higher among adolescents who were currently working, the differences were much smaller (4–9%) and not statistically significant (*p* > 0.05).

## 4. Discussion

A large proportion (29%) of participants had elevated blood pressure in this study of Hispanic adolescents, which was more common among males and those with chronic disease indicators (AN, overweight or obesity, and abdominal obesity). This is consistent with prior studies characterizing hypertension in children and adolescents [43,44]. A major strength of this study is that it is among the first to examine the potential impact of working on blood pressure in adolescents, specifically Hispanics. Working was a significant predictor of elevated blood pressure after adjusting for potential confounders at baseline. In addition, despite not being statistically significant, the mean probabilities of elevated blood pressure were higher for adolescents who were working. This association is also consistent with studies of adult working populations [24,25,26,27,28]. A detailed comparison with literature focusing on adults is difficult given differences in data collection and study variable definitions. The mechanism of how work exposures influence blood pressure could not be examined in this study. However, some of the participants in the study reported long work hours in addition to other demands related to school and home responsibilities. Insufficient time for engaging in healthy and stress relieving behaviors may be a factor. As an example, increased stress and limited time can negatively impact sleep quality and quantity. A recent systematic review indicated evidence of a link between sleep parameters and obesity, hypertension, and insulin sensitivity, but there were an insufficient number of high-quality studies available for assessing causality [45]. With respect to psychosocial job exposures, a study of Brazilian adolescent workers evaluated a connection between job demands, job control, and social support at work on various health indicators. The researchers found associations between psychological job demands and reduced sleep quantity during the week as well as work injury and body pain [46]. In the present study, self-reported sleep quantity was not associated with elevated blood pressure. The lack of association could be due to under or misreporting of sleep parameters since the data were self-reported. Furthermore, the outcome was elevated blood pressure rather than diagnosed hypertension. In addition, work-related as well as general stress may increase unhealthy coping behaviors in adolescents such as excessive food consumption, consuming foods higher in sugar and saturated fat, or consuming alcohol [30,31,32,33,34]. Many of these unhealthy coping mechanisms are associated with hypertension and coronary heart disease [47,48].

Additional strengths of this study included using an existing migrant education program infrastructure to access a hard-to-reach population [36]. Partnering closely with the school administrators and staff allowed for maintaining contact over time with a young, Hispanic population who are clearly at risk for chronic disease. Additional strengths included response proportions that were over 80% at baseline and during each assessment. Over 90% of eligible participants continued to participate in each follow-up assessment.

The key limitations of this study include a low prevalence of students currently engaged in work. While the use of pre-existing data made it possible to examine an under-researched topic with few resources, the low number of current workers also could have restricted the study’s statistical power, while also prohibiting the examination of specific types of work and work-related exposures, both physical and psychosocial. Examples of areas of interest for future studies include increased time demands and long work hours combined with other non-work demands on time, psychosocial job stress, and physical job exposures. Future research is needed that addresses other populations beyond Hispanics and that which is designed and powered specifically to examine specific work exposures within the context of nutritional and other risk factors. Another important limitation is a high rate of attrition due to school drop-out, relocating to another school district, and early graduation. Approximately 25% of those in the baseline assessment were no longer eligible to participant in the second follow-up assessment. A subsequent pilot study examined the impact of this loss to follow-up. Blood pressure levels were higher in those who dropped out; however levels were not significantly different when compared to those who remained in the study [36]. This loss to follow-up may have contributed to only a minimal average difference in elevated blood pressure levels comparing those currently working with those who were not in the follow-up assessments. Beyond the potential impact of loss to follow-up, the reason for the lack of an association across all years is not clear. It also could be that participants learned better skills for coping with stress and time demands as they aged. The variables collected for this study along with the sample size were not sufficient for examining these relationships further. The study design also did not allow diagnosis of high blood pressure in any single year. Guidelines recommend observing elevations in blood pressure on three separate occasions prior to making a diagnosis [10]. Finally, this study did not include an in-depth dietary assessment, which is a limitation given the known associations between diet and elevated blood pressure. This would be of particular concern if the working students ate a poorer diet than non-working students.

## 5. Conclusions

As CVD remains the leading cause of death in adults and the fifth leading cause of death for those aged 15–24 years old, there is a critical need for additional research and interventions to address CVD risk factors, such as high blood pressure [1,2,3]. In addition, as literature supports that CVD may originate during childhood or adolescence, it is vital to address these risk factors at this critical period [1,5,6,7,8]. In this study, working may have increased the risk of high blood pressure in adolescents, specifically those who are in their early high school years. Future studies based on a larger sample of workers are needed to confirm the present study’s findings and estimate the association between specific work-related exposures and elevated blood pressure, while accounting for nutritional and other potential risk factors. Future research could also identify the specific mechanism of how work status may increase hypertension in adolescents. The findings from this study provide a motivation for subsequent research in the areas of working youth and high blood pressure.

## Figures and Tables

**Table 1:** Baseline characteristics of participants by school.

Characteristics	Total (N = 508) ^a^	School 1 (N = 190)	School 2 (N = 318)	*p*-Value
	N (%)	N (%)	N (%)	
Gender				0.36 ^b^
Female	254 (50)	90 (47)	164 (52)	
Male	254 (50)	100 (53)	154 (48)	
Country of birth				0.95 ^b^
US	461 (92)	175 (92)	286 (92)	
Mexico or other	39 (8)	15 (8)	24 (8)	
Years lived in US				0.19 ^b^
<14	55 (11)	25 (13)	30 (9)	
≥14	453 (89)	165 (87)	288 (91)	
Work status in the prior year				0.01 ^b^
No work	298 (59)	128 (67)	170 (54)	
Any farm work	136 (27)	41 (22)	95 (30)	
Non-farm work only	72 (14)	21 (11)	51 (16)	
Working at the time of survey				0.15 ^b^
No	486 (96)	185 (97)	301 (95)	
Yes	22 (4)	5 (3)	17 (5)	
Television (TV) time on average school day				0.27 ^b^
None	87 (17)	28 (15)	59 (19)	
1+ hours	421 (83)	162 (85)	259 (81)	
Blood pressure				0.01 ^b^
Normal	360 (71)	147 (77)	213 (67)	
High or high normal	148 (29)	43 (23)	105 (33)	
Acanthosis nigricans				0.83 ^b^
No	385 (76)	143 (75)	242 (76)	
Yes	123 (24)	47 (25)	76 (24)	
Overweight or obese				0.07 ^b^
No	264 (52)	89 (47)	175 (55)	
Yes	244 (48)	101 (53)	143 (45)	
Waist at or above 75th percentile				0.05 ^b^
No	264 (52)	88 (47)	176 (56)	
Yes	240 (48)	100 (53)	140 (44)	
Experienced discrimination based on race or ethnicity				0.08 ^b^
Never	330 (65)	132 (70)	198 (62)	
Sometimes/often	177 (35)	57 (30)	120 (38)	
Depressive symptoms				0.97 ^b^
No	403 (80)	151 (80)	252 (80)	
Yes	102 (20)	38 (20)	64 (20)	
Number of sports teams in previous year				0.45 ^b^
None	250 (49)	97 (51)	153 (48)	
1–2 teams	206 (41)	77 (41)	129 (41)	
3+ teams	51 (10)	15 (8)	36 (11)	
Days being physically active				0.43 ^b^
None	70 (14)	32 (17)	38 (14)	
1–2 days	87 (17)	32 (17)	55 (17)	
3–6 days	222 (44)	77 (41)	145 (46)	
7 days	127 (25)	48 (25)	79 (25)	
**Continuous Variables**	**Mean (SD)**	**Mean (SD)**	**Mean (SD)**	***p*-Value**
Age (years)	15.0 (0.8)	14.9 (0.8)	15.0 (0.8)	0.25 ^c^
Body Mass Index (BMI)	25.8 (7.1)	26.5 (7.4)	25.4 (6.8)	0.10 ^c^
Average waist circumference (cm)	88.5 (17.4)	90.1 (17.7)	87.5 (17.1)	0.12 ^c^
Hours sleeping during weekdays	7.7 (1.3)	7.6 (1.4)	7.7 (1.3)	0.21 ^c^
Hours sleeping during weekend	8.9 (2.6)	8.8 (2.7)	9.0 (2.6)	0.50 ^c^

^a^ Characteristics may not sum to 508 due to missing values; ^b^ chi-square test; ^c^ t-test.

**Table 2:** Baseline characteristics of participants by blood pressure status.

Characteristics	School 1			School 2		
	Normal (N = 147)	High or High Normal (N = 43)		Normal (N = 213)	High or High Normal (N = 105)	
	N (%)	N (%)	*p*-Value	N (%)	N (%)	*p*-Value
Gender			0.01 ^a^			<0.0001 ^a^
Female	77 (52)	13 (30)		138 (65)	26 (25)	
Male	70 (47)	30 (70)		75 (35)	79 (75)	
Country of birth			0.80 ^a^			0.62 ^a^
US	135 (92)	40 (93)		193 (93)	93 (91)	
Mexico or other	12 (8)	3 (7)		15 (7)	9 (9)	
Years lived in US			0.86 ^a^			0.21 ^a^
<14	19 (13)	6 (14)		17 (8)	13 (12)	
≥14	128 (87)	37 (86)		196 (92)	92 (88)	
Work status in the previous year			0.44 ^a^			0.005 ^a^
No work	100 (68)	28 (65)		127 (60)	43 (41)	
Any farm work	33 (22)	8 (19)		53 (25)	42 (40)	
Non-farm work only	14 (10)	7 (16)		31 (15)	20 (19)	
Working at the time of survey						
No	145 (99)	40 (93)	0.08 ^b^	206 (97)	95 (90)	0.02 ^a^
Yes	2 (1)	3 (7)		7 (3)	10 (10)	
Television (TV) time on average school day			0.87 ^a^			0.60 ^a^
None	22 (15)	6 (14)		37 (17)	22 (21)	
1+ hours	125 (85)	37 (86)		176 (83)	83 (79)	
Acanthosis Nigricans			0.01 ^a^			0.01 ^a^
No	117 (80)	26 (60)		171 (80)	71 (66)	
Yes	30 (20)	17 (40)		42 (20)	34 (32)	
Overweight or obese			0.0004 ^a^			<0.0001 ^a^
No	79 (54)	10 (23)		134 (63)	41 (39)	
Yes	68 (46)	33 (77)		79 (37)	64 (61)	
Waist at or above 75th percentile			0.0007 ^a^			0.0005 ^a^
No	78 (53)	10 (24)		132 (63)	44 (42)	
Yes	68 (47)	32 (76)		79 (37)	61 (58)	
Experienced discrimination based on race or ethnicity			0.26 ^a^			0.93 ^a^
Never	99 (68)	33 (77)		133 (62)	65 (62)	
Sometimes/often	47 (32)	10 (23)		80 (38)	40 (38)	
Depressive symptoms			0.31 ^a^			0.23 ^a^
No	119 (82)	32 (74)		165 (78)	87 (84)	
Yes	27 (18)	11 (26)		47 (22)	17 (16)	
Number of sports teams in previous year			0.05 ^a^			0.03 ^a^
None	82 (56)	15 (35)		105 (49)	48 (46)	
1–2 teams	54 (37)	23 (53)		91 (43)	38 (36)	
3+ teams	10 (7)	5 (12)		17 (8)	19 (18)	
Days being physically active in the past 7 days			0.02 ^a^			0.06 ^a^
None	27 (18)	5 (12)		27 (13)	11 (10)	
1–2 days	26 (18)	6 (14)		38 (18)	17 (16)	
3–6 days	64 (44)	13 (30)		104 (49)	41 (39)	
7 days	29 (20)	19 (44)		43 (20)	36 (34)	
**Continuous Variables**	**Mean (SD)**	**Mean (SD)**	***p*-Value**	**Mean (SD)**	**Mean (SD)**	***p*-Value**
Age (years)	15 (0.8)	14.8 (0.7)	0.34 ^c^	14.9 (0.8)	15.2 (0.9)	0.01 ^c^
Body Mass Index (BMI)	25.7 (7.3)	28.9 (7.6)	0.01 ^c^	24.4 (6.4)	27.5 (7.1)	0.0001 ^c^
Average waist (cm)	88.3 (17.6)	96.0 (16.8)	0.01 ^c^	85.0 (16.4)	92.7 (17.5)	0.0002 ^c^
Hours sleeping during weekdays	7.6 (1.4)	7.3 (1.6)	0.23 ^c^	7.8 (1.3)	7.6 (1.3)	0.38 ^c^
Hours sleeping during weekend	9.0 (2.5)	8.2 (3.1)	0.08 ^c^	9.1 (2.6)	8.6 (2.4)	0.07 ^c^

^a^ chi-square test; ^b^ Fisher’s exact test; ^c^ t-test.

**Table 3:** Adjusted ^a^ mean probability of high or high normal blood pressure at each time point among students who were not working and who were currently working.

Assessment Year	Not Currently Working Mean (95% CI)	Currently Working Mean (95% CI)	Difference Mean (*p*-Value)
Baseline	0.24 (0.19, 0.29)	0.51 (0.29, 0.73)	0.27 (0.01)
Follow-up Year 1	0.24 (0.20, 0.29)	0.28 (0.15, 0.47)	0.04 (0.63)
Follow-up Year 2	0.32 (0.25, 0.39)	0.41 (0.23, 0.63)	0.09 (0.35)

^a^ Adjusted means from generalized linear mixed models with a logit link function, model covariates include: age, gender, AN, body mass index (BMI), number of days physically active in the past seven days, school participant enrolled, work status, year of survey, and the interaction of work status with year of survey.
